# Reviewer acknowledgement 2015

**DOI:** 10.1186/s13073-016-0283-2

**Published:** 2016-03-02

**Authors:** Rebecca F Furlong

**Affiliations:** BioMed Central, 236 Gray’s Inn Road, London, WC1X 8HB UK

## Abstract

The editors of *Genome Medicine* are extremely grateful for the time, hard work and support of all our reviewers, and would like to thank everyone who contributed to the journal in Volume 7 (2015).

**Christian Abnet**

USA

**Mark Adams**

USA

**Daniel Agardh**

Sweden

**Alexander Alekseyenko**

USA

**Emil Alexov**

USA

**Ana Alfirevic**

United Kingdom

**Fowzan Alkuraya**

Saudi Arabia

**Patrick Aloy**

Spain

**Patrick Arbuthnot**

South Africa

**Janelle Arthur**

USA

**Maxim Artyomov**

USA

**Euan Ashley**

USA

**Yan Asmann**

USA

**Gary Bader**

Canada

**Matthew Bainbridge**

USA

**Veerabhadran Baladandayuthapani**

USA

**Dennis Balligner**

USA

**Sergio Baranzini**

USA

**Anne Barton**

United Kingdom

**Katia Basso**

USA

**Stephan Beck**

United Kingdom

**Jeffrey Beekman**

Netherlands

**Robert Beiko**

Canada

**Andreas Bender**

United Kingdom

**Mikael Benson**

Sweden

**Michael Berger**

USA

**Ben Berkhout**

Netherlands

**Nancy Berliner**

USA

**Kapil Bharti**

USA

**Leslie Biesecker**

USA

**Joseph Blattman**

USA

**Cinnamon Bloss**

USA

**Cyril Bourgeois**

Italy

**Paul Boutros**

Canada

**James Brenton**

United Kingdom

**Alex Brodsky**

USA

**Angela Brooks**

USA

**Robert Brosh**

USA

**Matthew Brown**

Australia

**Eva Budínská**

Czech Republic

**Joel Buxbaum**

USA

**Daniel Cahill**

USA

**Mary Callanan**

France

**Raffaele Calogero**

Italy

**Fabien Campagne**

USA

**Colin Campbell**

United Kingdom

**Sally Camper**

USA

**Keşmir Can**

USA

**Ruaidhri Carmody**

United Kingdom

**Rita Casadio**

Italy

**Peter Castaldi**

USA

**Robert Castelo**

Spain

**Ulrich Certa**

Switzerland

**Kai-Ping Chang**

Taiwan

**Ken Chen**

USA

**Igor Chesnokov**

USA

**Michael Cho**

USA

**Dmitriy Chudakov**

Russian Federation

**Bekir Cinar**

USA

**Mete Civelek**

USA

**Sarah Cobey**

USA

**Lachlan Coin**

United Kingdom

**Paul Coleman**

USA

**Maria Carmen Collado**

Spain

**Philippe Collas**

Norway

**Andrew Collins**

Australia

**Peter Conlon**

United Kingdom

**Heather Cordell**

United Kingdom

**Sally Coulthard**

United Kingdom

**Mark Cowley**

Australia

**Jeffrey Craig**

Australia

**Xiangqin Cui**

USA

**Mehul Dattani**

United Kingdom

**Carsten Daub**

Sweden

**Paul De Bakker**

Netherlands

**Elza De Bruin**

United Kingdom

**Tom De Koning**

Netherlands

**Monica Barbosa De Melo**

Brazil

**Giada De Palma**

Canada

**Sandro De Souza**

Brazil

**Erick Denamur**

France

**Youping Deng**

USA

**Jorge Di Paola**

USA

**Dzung Diep**

Norway

**Todd Druley**

USA

**Desiree Du Sart**

Australia

**Eric Duncavage**

USA

**Deborah Dünn-Walters**

United Kingdom

**Bas Dutilh**

Netherlands

**Sian Ellard**

United Kingdom

**Laura Elo**

Finland

**Andrew Escayg**

USA

**Kilian Eyerich**

Germany

**Benjamin Fairfax**

United Kingdom

**Lindsey Farrer**

USA

**Jane Figueiredo**

USA

**Katja Fink**

Singapore

**Francesco Fiorentino**

Italy

**Darren Flower**

United Kingdom

**Anthony Fodor**

USA

**Alistair Forrest**

Japan

**Lude Franke**

Netherlands

**Wolfgang Florian Fricke**

USA

**Lars Fugger**

Denmark

**Birgit Funke**

USA

**Terry Furey**

USA

**France Gagnon**

Canada

**William Gallagher**

Ireland

**Jianjiong Gao**

USA

**Beatriz Garcia**

Spain

**Anne Gatignol**

Canada

**Nils Gehlenborg**

USA

**Sarah Gehlert**

USA

**Daniel Geraghty**

USA

**Clarissa Gerhauser**

Germany

**Marco Gerlinger**

United Kingdom

**Benjamin Gewurz**

USA

**Patrizio Giacomini**

Italy

**Greg Gibson**

USA

**Ajay Goel**

USA

**Tanya Golubchik**

United Kingdom

**Abel Gonzalez-Perez**

Spain

**Ellen Goode**

USA

**Jorg Goronzy**

USA

**Casey Greene**

USA

**Obi Griffith**

USA

**Kirsten Gronbaek**

Denmark

**Henk Jan An Guchelaar**

Netherlands

**Ian Hall**

United Kingdom

**Robin Hallett**

Canada

**Peter Hammerman**

USA

**Joshua Hare**

USA

**Reuben Harris**

USA

**Sampsa Hautaniemi**

Finland

**Stanley Hazen**

USA

**Shikun He**

USA

**Madhuri Hegde**

USA

**Kristin Hegstad**

Norway

**Jayne Hehir-Kwa**

Netherlands

**Zdenko Herceg**

France

**Pawel Herzyk**

United Kingdom

**Joshua Ho**

Australia

**Robert Hoehndorf**

United Kingdom

**Noah Hoffman**

USA

**Mattias Höglund**

Sweden

**Jill Hollenbach**

USA

**Monica Hollstein**

United Kingdom

**Mark Holmes**

United Kingdom

**Kathryn Holt**

Australia

**Robert Holt**

Canada

**David Hot**

France

**Zhenjun Hu**

USA

**Keqin Hua**

China

**Xiaojun Huang**

China

**Yun Huang**

USA

**Chi-Chung Hui**

Canada

**Lilia Iakoucheva**

USA

**Francesco Iorio**

United Kingdom

**Gregory Ippolito**

USA

**Laird Jackson**

USA

**Cecile Janssens**

USA

**Andrew Jarnicki**

Australia

**Evan Johnson**

USA

**Steven Jones**

Canada

**Philipp Kapranov**

USA

**Fredrik Karpe**

United Kingdom

**Tae-Min Kim**

Republic of Korea

**Stephen Kingsmore**

USA

**George Kirov**

United Kingdom

**Eric Klee**

USA

**Teri Klein**

USA

**Yuta Kochi**

Japan

**Isaac Kohane**

USA

**Sebastian Köhler**

Germany

**Rahul Kohli**

USA

**Sek Won Kong**

USA

**Genevieve Konopka**

USA

**R Frank Kooy**

Netherlands

**Sebastian Kreiter**

Germany

**Dietmar Krex**

Germany

**Leon Kuchenbecker**

Germany

**Ralf Kühn**

Germany

**Pia Kvistborg**

Netherlands

**Mikyoung Kwak**

Republic of Korea

**Marc Ladanyi**

USA

**Hugo Lam**

USA

**Erik Larsson**

Sweden

**Yuri Lebedev**

Russian Federation

**Su-In Lee**

USA

**Peter Lee**

USA

**Gaétan Lesca**

France

**Emil Lesho**

USA

**Ao Li**

China

**Yangqiu Li**

China

**Honghuang Lin**

USA

**Dan Littman**

USA

**Gang Liu**

USA

**Dajiang Liu**

USA

**Nick Loman**

United Kingdom

**Jacob Lorenzo-Moralez**

Spain

**Yoram Louzoun**

Israel

**Catherine Lozupone**

USA

**Qing Lu**

USA

**Gholson Lyon**

USA

**Ding Ma**

China

**Jian Ma**

USA

**Thomas Maccarthy**

USA

**Stuart Macgregor**

Australia

**Calum Macrae**

USA

**Christopher Maher**

USA

**Martin Maiers**

United Kingdom

**Marcus Mall**

Germany

**Angela Mally**

Germany

**Ilgar Mamedov**

Russian Federation

**Jelena Mann**

United Kingdom

**John Martens**

Netherlands

**William Martin**

USA

**Anuja Mathew**

USA

**Patrick Matthys**

Belgium

**Gabor Matyas**

Switzerland

**Manuel Mayr**

United Kingdom

**Gaston Mazandu**

South Africa

**Maria Cristina Mazzilli**

Italy

**Ramit Mehr**

Israel

**Peter Meikle**

Australia

**Alexander Mellmann**

Germany

**Fredrik Mertens**

Sweden

**Noriko Miyake**

Japan

**Peter Molloy**

Australia

**Lluis Montoliu**

Spain

**Yves Moreau**

Belgium

**Loris Mularoni**

Spain

**Ariel Munitz**

Israel

**Jeffrey N Myers**

USA

**June Myklebust**

Norway

**Niranjan Nagarajan**

Singapore

**Mike Nalls**

USA

**Giuseppe Narzisi**

USA

**Deborah Neklason**

USA

**Heather Nelson**

USA

**Anthony Nichols**

Canada

**Jens Nielsen**

Sweden

**Morten Nielsen**

Denmark

**Ruben Nogales-Cadenas**

USA

**Javier Ochoa-Repáraz**

USA

**Anne O'Donnell**

USA

**Mark O'Driscoll**

United Kingdom

**Justin O'Grady**

United Kingdom

**Heather O'Leary**

USA

**Stuart Orkin**

USA

**Alex Paciorkowski**

USA

**Sophie Paczesny**

USA

**Mark Pallen**

United Kingdom

**Jason Park**

USA

**Geraldo Passos**

Brazil

**Mary Patti**

USA

**John Paul**

United Kingdom

**Bruno Peault**

USA

**Jerry Pelletier**

Canada

**Stephen Pennington**

Ireland

**Bjoern Peters**

United States

**Sven Pettersson**

Sweden

**Minoli Perera**

USA

**Christine Petit**

France

**Roberto Pili**

USA

**Munir Pirmohamed**

United Kingdom

**Graham Plastow**

Canada

**Hendrik Poinar**

Canada

**Matthew Porteus**

USA

**Judy Potashkin**

USA

**James Priest**

USA

**Jeroen Raes**

Belgium

**Shoba Ranganathan**

Australia

**Mattias Rantalainen**

Sweden

**Benjamin Raphael**

USA

**Tobias Rausch**

Germany

**Sai Reddy**

Switzerland

**Jeff Reeve**

Canada

**Heidi Rehm**

USA

**Marcel Reinders**

Netherlands

**Sheila Reynolds**

USA

**Brigit Riley**

USA

**Laura Riva**

Italy

**Lawrence Rizzolo**

USA

**Paul Robbins**

USA

**Peter Robinson**

Germany

**Stephanie Roessler**

Germany

**Marco Roos**

Netherlands

**Richard Rosenquist**

Sweden

**Nicole Rosenzweig**

USA

**Theodora Ross**

USA

**Mark Rubin**

USA

**Wendy Rubinstein**

USA

**Serena Sanna**

Italy

**Deepak Saxena**

USA

**Eric Schadt**

USA

**John Schimenti**

USA

**Helgi Schioth**

Sweden

**Matthias Schwab**

Germany

**Cynthia Sears**

USA

**Ayellet Segrè**

USA

**Shamala Devi Sekaran**

Malaysia

**Tieliu Shi**

China

**Jung-Bum Shin**

USA

**Yuichi Shiraishi**

Japan

**Richard Simon**

USA

**Todd Skaar**

USA

**Bill Skarnes**

United Kingdom

**Heather Skirton**

United Kingdom

**Damian Smedley**

United Kingdom

**Gordon Smyth**

Australia

**Evan Snitkin**

USA

**Michael Snyder**

USA

**Harry Sokol**

France

**Artem Sokolov**

USA

**Erik Sonnhammer**

Sweden

**Rainer Spang**

Germany

**Michael Speicher**

Austria

**Nancy Spinner**

USA

**Arun Sreekumar**

USA

**Pramod Srivastava**

USA

**Justin Stebbing**

United Kingdom

**Adam Stevens**

United Kingdom

**Mark Sussman**

USA

**Mark Sutton**

United Kingdom

**David Tamborero**

Spain

**Aik-Choon Tan**

USA

**Mark Tanaka**

Australia

**Petrus Tang**

Taiwan

Republic Of China

**Peter Taschner**

Netherlands

**Graham Taylor**

United Kingdom

**Jenny Taylor**

United Kingdom

**Martin Taylor**

United Kingdom

**Sarah Teichmann**

United Kingdom

**Masanori Terajima**

USA

**Andrew Teschendorff**

United Kingdom

**Rhys Thomas**

United Kingdom

**Audrey Thurm**

USA

**Winston Timp**

USA

**Marianne Tinguely**

Switzerland

**Yigang Tong**

China

**Ali Torkamani**

USA

**James Traherne**

United Kingdom

**Zlatko Trajanoski**

Austria

**Stella Trompet**

Netherlands

**Christos Tsatsanis**

Greece

**George Tseng**

USA

**Peter Turnbaugh**

USA

**Jane Turton**

United Kingdom

**Svitlana Tyekucheva**

USA

**Eliezer Van Allen**

USA

**Anke Van Den Berg**

Netherlands

**Mariëlle Van Gijn**

Netherlands

**Fabio Vandin**

Denmark

**Joris Veltman**

Netherlands

**Vanessa Venturi**

Australia

**Gilles Vergnaud**

France

**David Vermijlen**

Belgium

**Claes Wahlestedt**

USA

**Haoyi Wang**

USA

**Qingguo Wang**

USA

**Wenyi Wang**

USA

**Yexun Wang**

USA

**Hedda Wardemann**

Germany

**Edus Warren**

USA

**Wyeth Wasserman**

Canada

**Robert Waterland**

United Kingdom

**David Waxman**

USA

**Benedikt Wefers**

Germany

**Bruce Weir**

USA

**Guido Werner**

Germany

**Robert West**

USA

**Peter White**

USA

**Marjolein Willemsen**

Netherlands

**Alexander Wilson**

USA

**Stacey Winham**

USA

**Naomi Wray**

Australia

**Caroline Wright**

United Kingdom

**Galen Wright**

Canada

**Masayuki Yamamoto**

Japan

**Mark Yandell**

USA

**Xia Yang**

USA

**Xia Yang**

China

**Esti Yeger-Lotem**

Israel

**Tsuyoshi Yokoi**

Japan

**Ken Young**

USA

**Haiyuan Yu**

USA

**Tianwei Yu**

USA

**Martin Zand**

USA

**Jiri Zavadil**

France

**Olga Zegarra-Moran**

Italy

**Georg Zeller**

Germany

**Michael Zemlin**

Germany

**Thorsten Zenz**

Germany

**Chi Zhang**

USA

**Luyong Zhang**

China

**Jianjun Zhang**

USA

**Kun Zhang**

USA

**Lu Zhen**

USA

**Yingye Zheng**

USA

**Jin Zhong**

China

**Jinfang Zhu**

USA

**Jun Zhu**

USA

**Baoli Zhu**

China

**Ivan Zyvagin**

Russian Federation

